# Dynamic Changes of Serum Heart Type-Fatty Acid Binding Protein in Cancer Patients Treated With Immune Checkpoint Inhibitors

**DOI:** 10.3389/fphar.2021.748677

**Published:** 2021-10-01

**Authors:** Ming Yuan, Li Zang, Aiqing Xu, Mengqi Gong, Qing Liu, Bin Huo, Jinhuan Wang, Huaying Fu, Gary Tse, Leonardo Roever, Guangping Li, Haitao Wang, Tong Liu

**Affiliations:** ^1^ Tianjin Key Laboratory of Ionic-Molecular Function of Cardiovascular Disease, Department of Cardiology, Tianjin Institute of Cardiology, Second Hospital of Tianjin Medical University, Tianjin, China; ^2^ Department of Cardiology, Biomedical Research (Therapy) Center, Sir Run Run Shaw Hospital, School of Medicine, Zhejiang University, Hangzhou, China; ^3^ Department of Oncology, Second Hospital of Tianjin Medical University, Tianjin, China; ^4^ Central Laboratory/Tianjin Research Institute of Urology, Second Hospital of Tianjin Medical University, Tianjin, China; ^5^ Department of Clinical Research, Federal University of Uberlândia, Uberlândia, Brazil

**Keywords:** cardio-oncology, cardiotoxicity, immune checkpoint inhibitors, H-FABP, immune-related adverse events

## Abstract

**Objective:** Immune checkpoint inhibitors (ICIs) are effective anti-cancer drugs that can improve survival in cancer patients, but their use may be associated with adverse cardiovascular side effects. Therefore, there is a clinical unmet need to identify non-invasive biomarker to detect subclinical cardiac toxicity after ICI treatment. The aim of this study is to examine the plasma levels of biomarkers in cancer survivors who were treated with ICIs.

**Patients and Methods:** In a cohort of 19 cancer patients, biomarkers were evaluated at baseline, 1 month, 3 and 6 months after ICI administration. These biomarkers, hypothesized to be mechanistically relevant to cardiotoxicity, included cardiac troponin I (cTnI), high-sensitivity C-reactive protein (Hs-CRP), N-terminal pro–B-type natriuretic peptide (NT-pro BNP), CK (creatine kinase), CK-MB (creatine kinase-MB), Pentraxin-related protein 3 (PTX3), growth differentiation factor 15 (GDF-15), heart type-fatty acid binding protein (H-FABP) and galectin 3 (Gal-3).

**Results:** H-FABP, but not other biomarkers, were increased at 3 months, which persisted at 6 months (529.28 ± 312.83 vs. 752.33 ± 283.65 vs. 808.00 ± 289.69 pg/ml, *p* = 0.031 and *p* = 0.013). Left ventricular ejection fraction (63.00 ± 4.15% vs. 63.74 ± 4.07%, *p* > 0.05) was not significantly reduced at this time point.

**Conclusions:** H-FABP, but not other biomarkers, were increased in patients who were treated using ICIs. H-FABP might be a more sensitive biomarker to detect ICI-related subclinical myocardial damage than traditional cardiac biomarkers.

## Introduction

Utilizing the immune system to recognize and target malignant cells has been a strategy used in oncology for many years (Thomas and Hassan, 2012). In the past decades, a major advancement has been the use of immune checkpoint inhibitors (ICIs) to block antibodies expressed on T lymphocytes, including cytotoxic T-lymphocyte-associated protein-4 (CTLA-4) and programmed cell death protein-1 (PD-1) (Ansell et al., 2015; Wolchok et al., 2017), or their corresponding ligands expressed on tumor cells, such as programmed cell death 1 ligand-1 (PD-L1) (Herbst et al., 2016; Gulley et al., 2017; Choueiri et al., 2018). ICIs including CTLA-4 (ipilimumab), PD-1 (nivolumab or pembrolizumab), and PD-L1 (atezolizumab, avelumab, or durvalumab), either as a monotherapy or combination therapy can induce tumor responses in melanoma, non-small cell lung cancer (NSCLC), renal cell cancer (RCC), head and neck squamous cell carcinoma, urothelial cancer, breast cancer and Hodgkin disease (Ribas and Wolchok, 2018). Whilst the use of ICIs has led to improvements in cancer survival, however, the suppression of immune responses producing a range of autoimmune adverse events. The most common immune-mediated adverse events in patients receiving ICIs are fatigue, rashes, diarrhea, colitis, pulmonary and renal systems were reported (Postow et al., 2018). These agents also carry a significant risk of cardiovascular morbidity, including LVEF decline, cardiomyopathy, and HF (Voskens et al., 2013; Wang et al., 2018). Myocarditis is an infrequent but potential fatal complication of ICIs. Myocarditis with clinical presentations ranging from asymptomatic cardiac biomarker elevation to heart failure, arrhythmia, cardiac fibrosis and pericarditis (Gibson et al., 2016; Mahmood et al., 2018; Neilan et al., 2018; Altan et al., 2019). Another study found that the incidence of myocarditis is 1.14% in a cohort of cancer patients treated with ICIs (Mahmood et al., 2018). Echocardiography and electrocardiography are routinely used but they show low sensitivity for the early prediction of myocardial damage. Whilst endomyocardial biopsy has the degree of specificity and sensitivity needed for diagnosing ICI-associated myocarditis, it is considered to be too invasive and expensive for serial monitoring. Therefore, there is an unmet clinical need to find useful diagnostic tools for the early identification or accurate prediction of those who may be suffering from ICI-related myocarditis. Early identification and preemptive cardioprotective treatment can reduce interruptions or discontinuations in cancer therapy, and reduce cardiovascular burden and potentially maximize the tolerable doses of ICIs to improve cancer-related mortality. The aim of this study is to investigate the changes of the plasma levels of biomarkers in cancer survivors who were treated with ICIs with a follow-up period of 6 months. The biomarkers reflected cardiomyocyte injury [cardiac troponin I (cTnI), CK (creatine kinase), CK-MB (creatine kinase-MB)]; inflammation [high-sensitivity C-reactive protein (Hs-CRP), Pentraxin-related protein (PTX3) and growth differentiation factor 15 (GDF-15)]; neurohormonal activation [N-terminal pro–B-type natriuretic peptide (NT-pro BNP)]; heart type-fatty acid binding protein (H-FABP); and fibrosis [galectin 3 (Gal-3)]Their associations with myocardial damage, occurring either concurrently or at a subsequent visit were determined.

## Methods

### Study Population

The study protocol was approved by the institutional review boards of the participating institutions. The study is registered with the Chinese Clinical Trial Registry (registration number: ChiCTR18000162216). All participants provided informed consent. A total of 19 consecutive patients who never used the ICIs and hospitalized in the Oncology Department of Tianjin Medical University Second Hospital from June 2018 to June 2019 were enrolled to the study. Eligible patients were 18–85 years of age, diagnosed with cancer and planned for adjuvant therapy with ICIs. Patients with one of the following conditions were excluded: intolerance to ICIs treatment; severe left main coronary artery disease confirmed by angiography; presence of grade III or IV heart failure; acute myocardial infarction (acute or recovery period); hepatic insufficiency (AST/ALT >80U/L); severe renal insufficiency (eGER ≤ 30 m/min/1.73 m^2^); thyroid disease; acute infectious diseases; mental illness; life expectancy is less than 6 months.

At baseline (before immune-therapy) and 1month, 3 months, 6 months after ICIs administration, demographics and clinical history were recorded. Transthoracic echocardiograms were obtained at each visit, and blood samples were obtained immediately before cancer therapy infusion at 1, 3, 6-month intervals.

### Echocardiography

A transthoracic echocardiographic examination was performed in all patients using the Vivid-7 system equipped with a 2.4 MHz transducer (GE Medical Systems, Milwaukee, WI, USA). Left ventricular end systolic diameter (LVEDS), left ventricular end-diastolic diameter (LVEDD) and left ventricular ejection fraction (LVEF) were obtained using Simpson’s method of discs in the apical 4- and 2-chamber views as recommended by the American Society of Echocardiography. All tracings were made by a single observer and the data measurement is done by two ultrasound doctors at a centralized reading center who was blinded to all other clinical or biomarker data.

### Biomarkers

At the time of the echocardiography, venous blood samples were drawn from all participants and collected in standard tubes for plasma with EDTA. Place the collected blood samples properly, it is strictly forbidden to freeze whole blood to avoid hemolysis. After the blood culture sampling is completed, it should be sent to the biological laboratory for verification by a dedicated person immediately, generally not more than 2 h hemolysis specimens centrifuged at 3,000 rpm for 15 min, plasma was removed, allocated, and frozen at −80°C until assay. Measurement of cTnI, NT-proBNP, CK, CK-MB, Hs-CRP were performed on the clinical Laboratory System. Serum levels of GDF-15, Gal-3, PTX3 and H-FABP were assayed using commercially available ELISA kit (Cusabio Biotech Corporation, China). All measurements were performed on previously unthawed samples. Concentrations of unknown samples are determined using a standard curve that was constructed by plotting absorbance values against concentrations of the standards.

### Statistical Methods

Each biomarker was evaluated at baseline, 1 month, 3 and 6 months. The normality of the distribution of each continuous variable was tested by the Kolmogorov–Smirnov test. Continuous variables were reported as means ± SD or median (interquartile range) as appropriate. Statistical analysis was performed using the ANOVA test for continuous variables. A *p* value of <0.05 was regarded as statistically significant. All tests were two tailed and analyses were performed using SPSS 22.0 Statistical Package Program (SPSS Inc., Chicago, IL, United States).

## Results

### Patients Population

The baseline characteristics of the study cohort are shown in Table 1. The mean age of patients (*N* = 19) who using ICIs was 48.2 ± 11.6 years with 42.1% being male. Prior to ICIs administration, only one patient had previous myocardial infarction and coronary stenting, but the left ventricular ejection fraction (LVEF) was >50% in all those with a baseline measurement.

**TABLE1:** Baseline Characteristics of study participants.

	All participants *N* = 19
Age at start of ICI,yrs	48.2 ± 11.6
Male	8 (42.1)
Body mass index, kg/m2	21.3 ± 2.7
Fasting glucose	5.47 ± 0.77
Medical History and Risk Factors	
History of hypertension	5 (26.3)
History of diabetes	4 (21.1)
Tobacco use	
Current	2 (10.5)
Former	2 (10.5)
Alcohol intake	6 (31.6)
Hypercholesterolemia	2 (10.5)
Prior myocardial infraction	1 (5.3)
Prior coronary stenting	1 (5.3)
Prior atrial fibrillation	0 (0)
Prior COPD	0 (0)
Prior chronic kidney disease	2 (10.5)
Primary Cancer Type	
Lung cancer	3 (15.8)
Rectal cancer	1 (5.3)
Female reproductive system tumor	4 (21.1)
Bladder Cancer	1 (5.3)
Breast cancer	2 (10.5)
Renal cell carcinoma	4 (10.5)
Melanmoa	1 (5.3)
Other	3 (15.8)
Pre-ICI home CV Medications	
Beta-blockers	2 (10.5)
Aspirin	1 (5.3)
Statin	1 (5.3)
Calcium-channel blocker	3 (15.8)
ACE inhibitors or ARBs	2 (10.5)
Current monotherapy ICI	
Nivolumab (anti-PD1)	10 (52.6)
Sintilimab (anti-PD1)	2 (10.5)
Durvalumab (anti-PDL1)	7 (36.8)
ICI combined chemotherapy or radiation	
Radiation	2 (10.5)
Bevacizumab	1 (5.3)
VEGF inhibitors	3 (15.8)
Other chemotherapy	3 (15.8)
Other immune side effects during treatment	
No other immune side effects	15 (78.9)
Thyroiditis	2 (10.5)
Iritis	1 (5.3)
Colitis	1 (5.3)

Values are mean ± SD or n (%). Abbreviations: ICI = immune checkpoint inhibitors; CV = cardiovascular; ACE = Angiotensin-converting enzyme; ARBs = Angiotensin receptor blocker; COPD: chronic obstructive pulmonary diseases; anti-PD1: anti-programmed cell death protein 1; VEGF inhibitors: vascular endothelial growth factor inhibitors.

### Indications and Side Effects of ICIs

The most common indications for ICIs were Renal cell carcinoma, lung cancer and female reproductive system tumor. Of the monotherapy at the time of presentation, anti-PD1 was the most frequent (63.1%) in cases. For better therapeutic effect and clinical prognosis, single ICI therapy administrated with radiation or other chemotherapy is presented in Table 1. More than one-half (78.9%) of the cases had not experienced other ICI-related side effect.

Cardiac function and biomarkers at 1, 3, 6 months after ICI therapy compared to baseline.

**FIGURE 1:**
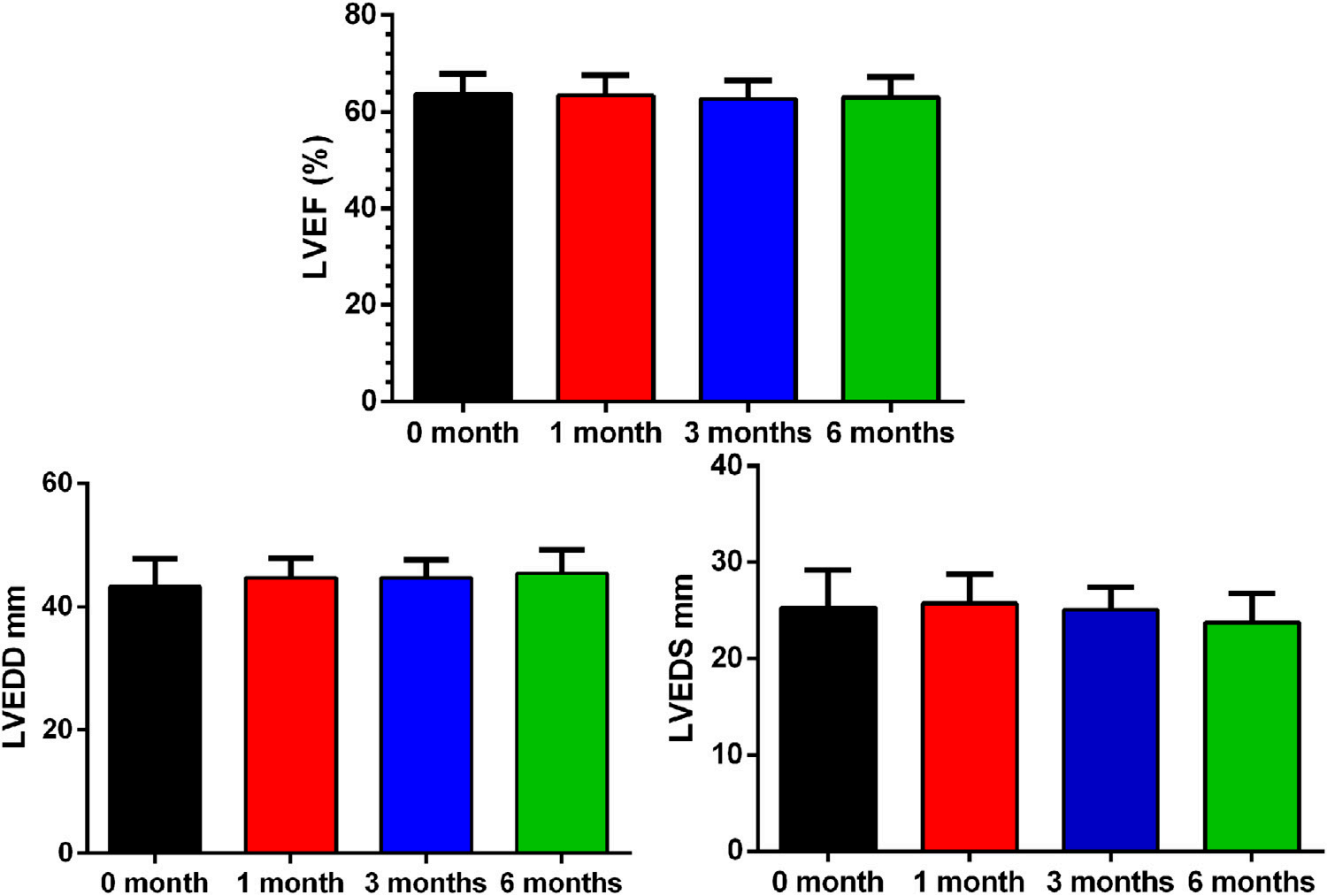
Baseline and Interval Changes in Echocardiography. LVEF: left ventricular ejection fraction; LVEDD: left ventricular end diastolic diameter.

There were no myocarditis cases and other clinical adverse cardiovascular events within 6 months of starting therapy. Compared to baseline, there was no change in LVEF (*p* = 0.608), LVEDD (*p* = 0.105) or LVEDS (*p* = 0.178) 6 months after ICI therapy. (Figure 1; Table 2).

**TABLE 2:** Baseline and Interval Changes in Echocardiography and Biomarker Levels Between Baseline and 1, 3 and 6 months.

	0 month	1 month	3 months	6 months
LVEF%	63.74 ± 4.07	63.41 ± 4.23	62.69 ± 3.79	63.00 ± 4.15
LVEDS mm	25.28 ± 3.93	25.73 ± 3.04	25.05 ± 2.37	23.76 ± 2.99
LVEDD mm	43.29 ± 4.46	44.74 ± 3.14	44.71 ± 2.98	45.43 ± 3.86
cTnI ng/ml	0.001 (0.001–0.002)	0.001 (0.001–0.0025)	0.0015 (0.001–0.002)	0.001 (0.001–0.001)
NT-proBNPng/L	82.30 (61.20–87.50)	64.00 (41.25–99.35)	79.45 (36.17–128.25)	147.00 (40.50–208.50)
D-dimer ng/ml	791.64 (478.43–2,187.79)	506.04 (430.01–1,042.59)	538.35 (303.43–925.55)	416.38 (297.23–938.05)
CK U/L	43.40 (34.70–88.90)	46.80 (37.00–87.00)	59.20 (34.52–85.12)	104.60 (50.55–177.37)
CK-MB U/L	17.16 ± 6.92	16.68 ± 5.79	18.01 ± 5.97	16.89 ± 11.42
Hs-CRP mg/L	10.08 (5.26–51.22)	7.98 (2.57–23.28)	9.28 (2.10–16.62)	3.71 (2.36–23.37)
GDF-15 pg/ml	1,075.85 ± 841.00	934.19 ± 623.22	870.14 ± 694.02	389.57 ± 199.74*
Gal-3 ng/ml	613.25 (462.99–957.24)	556.07 (428.21–759.64)	472.35 (443.52–540.28)	470.77 (439.40–536.31)
PTX3 pg/ml	398.82 ± 297.96	224.54 ± 143.58*	253.69 ± 166.15	310.02 ± 116.86
H-FABP pg/ml	529.28 ± 312.83	666.76 ± 187.87	752.33 ± 283.65*	808.00 ± 289.69*
Scr umol/l	62.42 ± 24.75	68.53 ± 21.90	65.54 ± 21.42	61.88 ± 14.63

Abbreviations: LVEF: left ventricular ejection fraction; LVEDS: left ventricular end systolic diameter; LVEDD: left ventricular end diastolic diameter; cTnI: cardiac troponin I; NT-proBNP: N-terminal pro–B-type natriuretic peptide; CK: creatine kinase; Hs-CRP: high-sensitivity C-reactive protein; GDF-15: growth differentiation factor 15; Gal-3: galectin 3; PTX3: pentraxin-related protein 3; H-FABP: heart type-fatty acid binding protein. Values are mean ± SD or median (interquartile range); * was compared with the 0 month group, P < 0.05

**FIGURE 2:**
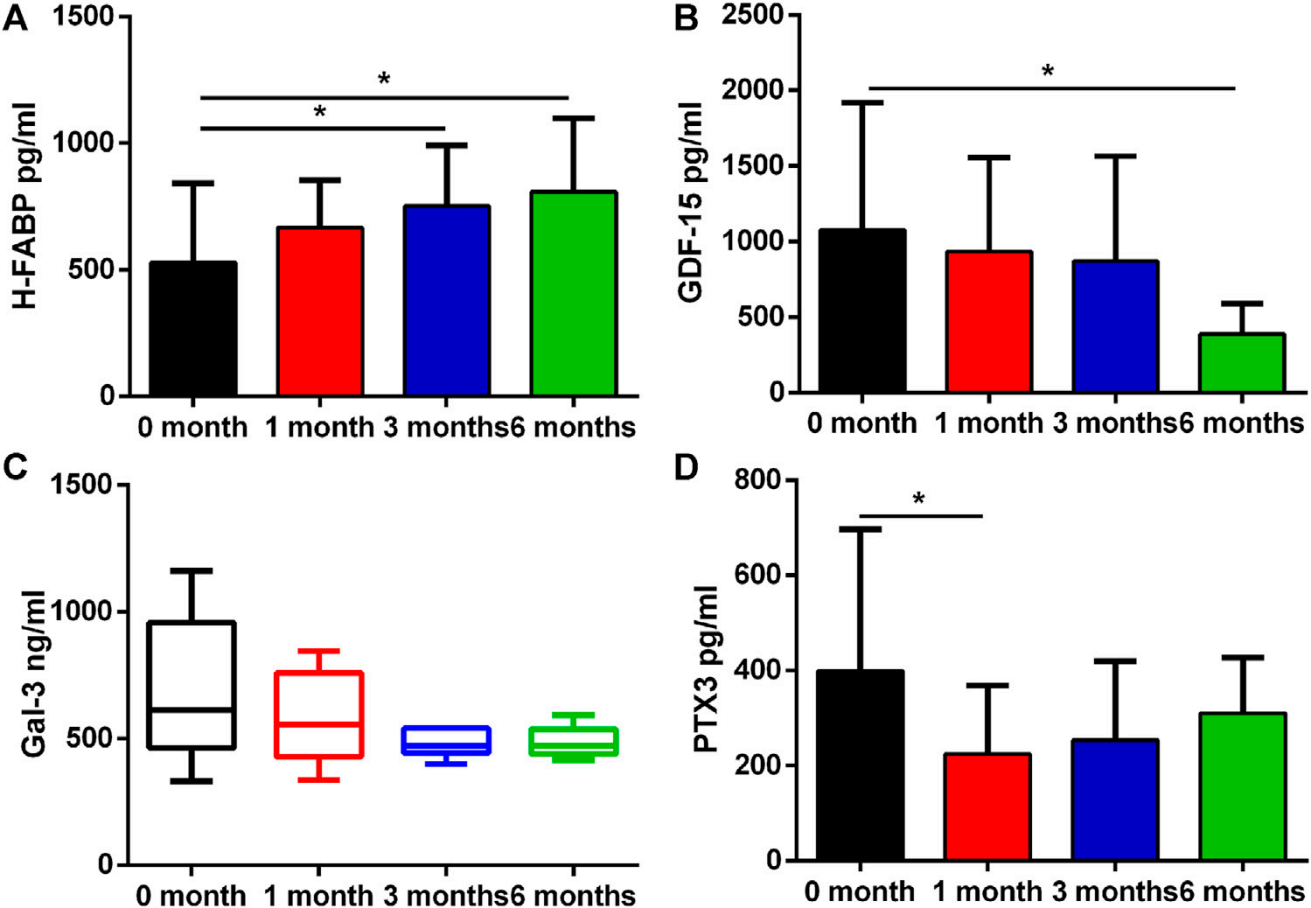
Proteins that had differential plasma levels between baseline and 1, 3 and 6 months. Edges of boxes denote 25th and 75th percentiles, lines are median concentrations, and error bars are minimum and maximum concentrations. **(A)** NT-pro BNP: N-terminal pro–B-type natriuretic peptide; **(B)** D-dimer; **(C)** CK: creatine kinase; **(D)** CK-MB: creatine kinase-MB.

**FIGURE 3:**
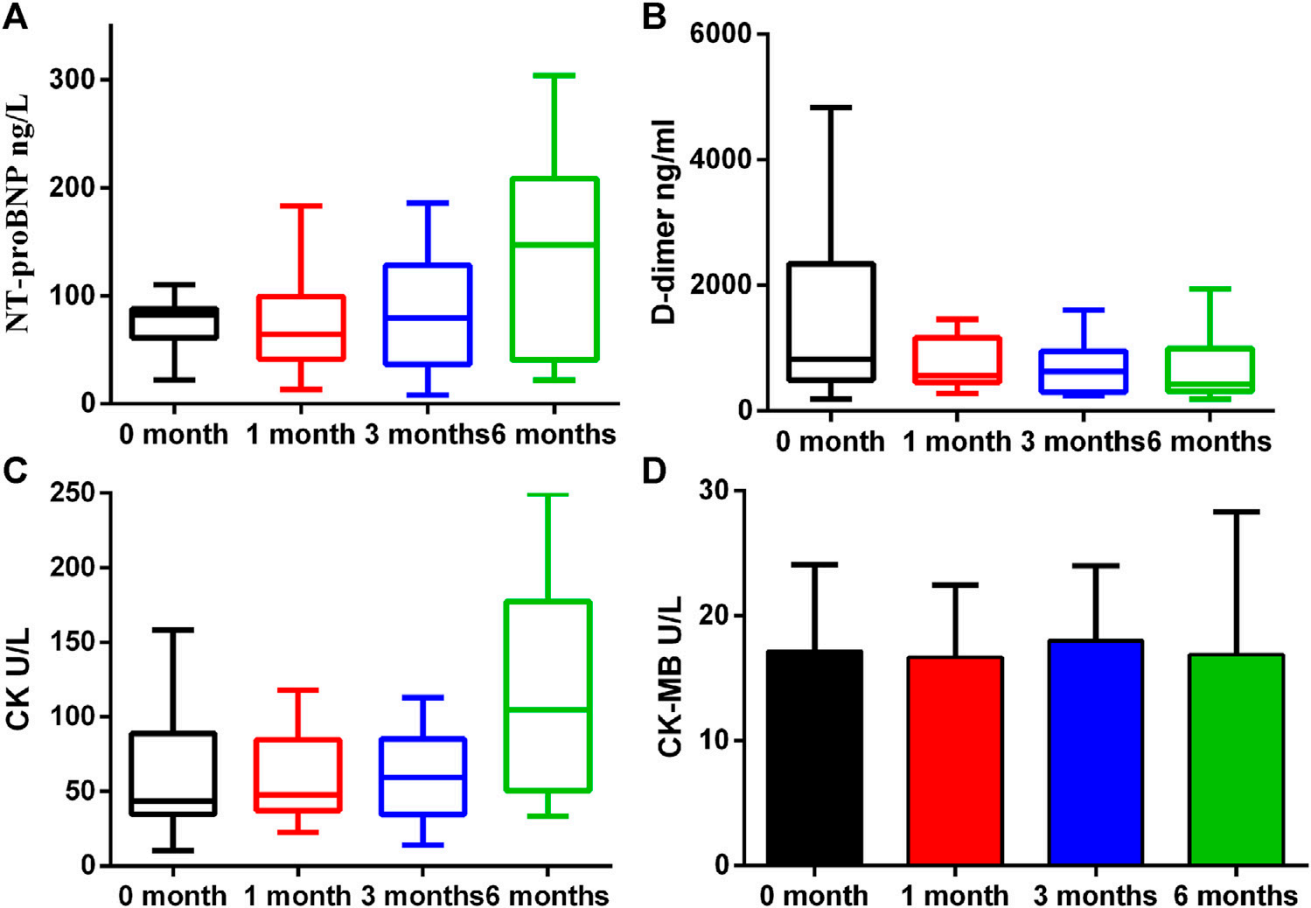
Plasma concentration changes of H-FABP, GDF-15, Gal-3, and PTX3 over ICIs treatment time. Edges of boxes denote 25th and 75th percentiles, lines are median concentrations, and error bars are minimum and maximum concentrations. **(A)** H-FABP: heart type-fatty acid binding protein; **(B)** GDF-15: growth differentiation factor 15; **(C)** Gal-3: galectin 3; **(D)** PTX3: pentraxin-related protein 3.

A number of biomarkers were evaluated before and after initiation of ICI therapy at different timepoints (Table 2). H-FABP levels were significantly elevated at 3 months which persisted at 6 months (*p* = 0.031 and *p* = 0.013), whereas GDF-15 was significantly lower only at 6 months (*p* = 0.017). Gal-3 was not significantly altered whilst PTX3 was significantly reduced at 1 month (*p* = 0.025, Figure 2). By contrast, no significant changes in cTnI (*p* = 0.24), D-dimer (*p* = 0.219), CK (*p* = 0.059) and CK-MB (*p* = 0.92) were observed at 1 month, 3 and 6 months (Figure 3).

## Discussion

The main findings of this prospective cohort study are that H-FABP levels, but not other biomarkers, were increased at three months in an absence of detectable reductions in LVEF.

ICIs have shown important benefits for improved treatment of several cancers, having demonstrated high response rates and prolonged overall survival in cancer patients. In 2015, the United States are almost 590,000 patients who are indicated for ICIs therapy (Andrews, 2015), and the ICIs market will experience considerable growth over the coming period (Webster, 2014). Nevertheless, the incidence of early and late adverse events associated with ICIs is unknown. ICIs are known to upregulate T-cell-mediated immune responses against cancer cells, although adverse events are a common occurrence, cardiotoxic effects are infrequent but are often associated with high mortality (Lyon et al., 2018). The immunotherapy trial data from Bristol-Myers Squibb found 18 cases (0.09%) of myocarditis among 20,594 subjects in the pharmacovigilance database. The incidence of myocarditis was higher in patients receiving a combination of nivolumab and ipilimumab (0.27%) than in those receiving nivolumab alone (0.06%) (Johnson et al., 2016). A multi-center registration study of 8 medical centers showed that the incidence of myocarditis was 1.14% (Mahmood et al., 2018). The median time from the application of ICIs to the onset of myocarditis was 34 days, and the age of patients with myocarditis was 65 ± 13 years, of which 29% were women. Compared with the control group, patients with combined ICIs (34 vs. 2%) and diabetes (34 vs. 13%) had a higher incidence of myocarditis. Our results suggest that individual biomarkers may show early changes that can reflect subclinical cardiac dysfunction following use of ICIs.

Fatty acid-binding proteins (FABPs) are relatively small cytoplasmic proteins that are abundant in tissues with active fatty acid metabolism, including the heart and the skeletal muscles. In fact, heart-type FABP (H-FABP) is particularly important for cardiomyocyte energy homoeostasis, since 50–80% of the heart’s energy is provided by the oxidation of fatty acids and H-FABP ensures intracellular transport of insoluble fatty acids. Following myocardial cell damage, H-FABP diffuse much more rapidly than troponins secretion into the interstitial space and appears in the circulation as early as 90 min after the onset of symptoms. Recently, H-FABP has become a newer perspective marker for the early detection of myocardial ischemia and necrosis, evaluated in the diagnostics and risk stratification of acute coronary syndromes (OʼDonoghue et al., 2006; Viswanathan et al., 2010; Hai-Long et al., 2018). Experimentally, concentrations of H-FABP, as measured by the MSD immunoassay, have been found to increase in rats treated with various myotoxic compounds (Tonomura et al., 2012). ElGhandour at al. studied 40 non-Hodgkin’s lymphoma patients treated with 6 cycles of cardiotoxic chemotherapy containing Doxorubicin (DOX). The authors concluded that H-FABP may serve as a reliable early predictor of cardiomyopathy induced by DOX. Our findings suggest that there is an association between H-FABP increase and subsequent myocardial dysfunction and provide evidence to support the importance of assessing changes in biomarkers over time. The utility of H-FABP appears to lie in evaluating levels after initial ICIs therapy exposure as opposed to baseline. To our knowledge, we describe for the first time an association between early changes in H-FABP levels and subsequent subclinical myocardial damage induced by treatment with ICIs. The heart-type-Fatty-Acid-Binding-Protein (H-FABP) is a protein, which is involved in intracellular myocardial transport. After myocardial necrosis H-FABP is rapidly released into the blood stream and was therefore investigated as a biomarker for acute myocardial infarction (AMI). In the study, H-FABP was detected to determine the potential utility for the early identification of cancer patients at risk for ICI-associated subclinical myocardial damage. Whilst H-FABP is a biomarker of cardiac dysfunction or damage, ICIs may cause systemic side effects that can lead to abnormal levels of this protein.

Gal-3 is a 26-kDa protein that is expressed by macrophages, plays a prominent role in tumor growth, metastasis, angiogenesis, and immune evasion and is believed to be a mediator of profibrotic pathways, stimulating cardiac fibroblasts to proliferate and deposit collagen. Gal-3 concentrations are elevated in patients with acute HF and are predictive of an increased risk of adverse outcomes (Shah et al., 2010). On the other hand, Gal-3 is highly expressed within the tumor microenvironment of aggressive cancers and whose expression correlates with poor survival particularly in patients with cancer (Vuong et al., 2019).

PTX3 is a member of the pentraxin family, the prototype of the pentraxin family is C reactive protein (CRP), a widely used clinical biomarker in human pathologies with an inflammatory or infectious origin (Lee et al., 2018). Nowadays, inflammation is considered an important determinant of cardiovascular disease and related risk factors including diabetes, hypertension (Libby, 2012). Among different inflammatory molecules, cytokines such as tumor necrosis factor (TNF)-α, interleukin (IL)-1β, and IL-6 were suggested pathophysiological players. Plasma PTX3 levels are elevated in patients with unstable angina pectoris (Inoue et al., 2007) and in patients undergoing coronary stenting (Kotooka et al., 2008). In heart failure, the plasma PTX3 level provides important prognostic information for the risk stratification of patients (Suzuki et al., 2008). GDF-15 is a member of the TGF-β cytokine superfamily that is produced in response to oxidative stress, inflammation, and injury (Wollert et al., 2007) and is an emerging marker in acute coronary syndromes and HF. The mechanisms of ICIs associated cardiotoxicity is unknown, and we found no significant association between either GDF-15 or PTX3 and ICI-associated subclinical myocardial damage. Irena et al. (Kaplanov et al., 2019) indicated that the particular importance to tumor-mediated inflammation is IL-1β, IL-1β is increased in most cancers, including breast cancer in advanced stages, where it is mainly expressed by cells in the microenvironment and enhances progression and metastasis. The reduction of inflammatory markers, such as GDF-15 and PTX3, in this study may be linked to increased survival and response to immune therapy. Further studies should explore the relationship between different biomarkers and outcomes after immune therapy.

### Study Limitation

There are several limitations of this study. Firstly, a relatively small number of patients was enrolled. Further studies in a larger number of patients will be needed to define the potential role of new circulating biomarkers in the assessment of ICIs-induced subclinical myocardial injury. Secondly, follow-up was relative short at 6 months. Thirdly, myocarditis is more prevalent in patients with combined use of CTLA-4 and PD-1/PD-L1, but CTLA-4 has not yet been marketed in the Mainland and was therefore not included in this study. Lastly, the application of imaging such as cardiac magnetic resonance imaging (CMR) or speckle-tracking echocardiography would be able to quantify the degree and location of cardiac dysfunction more precisely. had been resonated, the accuracy of the diagnosis could be improved. For example, speckle tracking imaging is recommended by ESC for detecting subclinical cardiac damage. It can be used in combination with measurements of H-FABP levels for further patient monitoring and risk stratification.

## Conclusion

H-FABP, but not other biomarkers, were increased in patients underwent ICIs therapy. H-FABP might be a more sensitive biomarker to detect ICI-related subclinical myocardial damage than traditional cardiac biomarkers.

## Data Availability

The raw data supporting the conclusion of this article will be made available by the authors, without undue reservation, to any qualified researcher.
